# Quantum sensing of broadband spin dynamics and magnon transport in antiferromagnets

**DOI:** 10.1126/sciadv.adu9381

**Published:** 2025-06-27

**Authors:** Alex L. Melendez, Shekhar Das, Francisco Ayala Rodriguez, I-Hsuan Kao, Wenhao Liu, Archibald J. Williams, Bing Lv, Joshua Goldberger, Shubhayu Chatterjee, Simranjeet Singh, P. Chris Hammel

**Affiliations:** ^1^ Department of Physics, The Ohio State University, Columbus, OH 43210, USA.; ^2^ Department of Physics, Carnegie Mellon University, Pittsburgh, PA 15213, USA.; ^3^ Department of Physics, The University of Texas at Dallas, Richardson, TX 75080, USA.; ^4^ Department of Chemistry and Biochemistry, The Ohio State University, Columbus, OH 43210, USA.

## Abstract

Optical detection of magnetic resonance using quantum spin sensors (QSSs) provides a spatially local and sensitive technique to probe spin dynamics in magnets. However, its utility as a probe of antiferromagnetic resonance (AFMR) remains an open question. We report the experimental demonstration of optically detected AFMR in layered van der Waals antiferromagnets (AFM) up to frequencies of 24 gigahertz. We leverage QSS spin relaxation due to low-frequency magnetic field fluctuations arising from collective dynamics of magnons excited by the uniform AFMR mode. First, through AFMR spectroscopy, we characterize the intrinsic exchange fields and magnetic anisotropies of the AFM. Second, using the localized sensitivity of the QSS, we demonstrate magnon transport over tens of micrometers. Last, we find that optical detection efficiency increases with increasing frequency. This showcases the dual capabilities of QSS as detectors of high-frequency magnetization dynamics and magnon transport, paving the way for understanding and controlling the magnetism of antiferromagnets.

## Teaser

Color centers break the speed limit to enable broadband quantum sensing of high-frequency antiferromagnetic dynamics.

## INTRODUCTION

Magnetic resonance is a spectroscopically precise tool capable of probing spin interactions and revealing detailed aspects of magnetic dynamics. The understanding of spin dynamics in antiferromagnets (AFMs), still in its infancy, is essential to realize conceptualized spintronic devices for the generation, transmission, and detection of high-frequency signals (*1*, *2*). In recent years, van der Waals (vdW) AFMs such as CrCl_3_ and CrSBr (*3*–*11*) have emerged as a promising platform for studying spin dynamics. Compared to traditional AFMs such as metal oxides or fluorides, the smaller antiferromagnetic exchange in vdW AFMs allows experimentation to probe the fundamentals of antiferromagnetic spin dynamics at relatively lower frequencies. This calls for new experimental tools that are able to provide a sensitive and spatially localized measurement of spin dynamics of AFMs over a broad frequency spectrum. Developing and using such tools will be central to understanding emergent dynamical phenomena in AFMs for spintronic applications.

In recent years, color defects in solids, such as nitrogen vacancy (NV^−^) centers in diamond, have emerged as sensitive optical probes enabling optical detection of ferromagnetic resonance and hence magnetic dynamics, through their coupling to the fluctuating stray magnetic fields generated by magnons in ferromagnets (*12*–*14*). The NV^−^ center is an atomic-scale quantum spin sensor (QSS) whose spin-dependent photoluminescence locally probes static and dynamic stray fields (*15*–*17*). In this role, its sensitivity to magnetization dynamics arises from the enhancement of the QSS spin relaxation rate by the fluctuating fields emanating from the magnon gas in the sample, specifically those magnetic field fluctuations matching the QSS electron spin resonance (ESR) frequency $\omega_{\text{NV}}$ . However, an experimental demonstration of the utility of the NV^−^ center for optical detection of magnetic resonance in AFMs, i.e., Optically Detected Anteferromagnetic Resonance (ODAFMR), wherein the characteristic resonant frequencies can range from a few gigahertz to hundreds gigahertz, is critically missing.

Here, we provide an experimental demonstration of the detection of ODAFMR, using NV^−^ centers as QSSs, in two vdW AFMs: CrCl_3_ and CrSBr. Our main results are as follows. First, we illustrate the effectiveness of the QSS relaxometric method through temperature- and magnetic field–dependent optical measurements of the magnetic resonance modes of CrCl_3_ and CrSBr and extract the intrinsic exchange fields and magnetic anisotropies from the ODAFMR spectra. Our results are consistent with previous measurements that use conventional inductive methods (*7*, *8*). Next, we show that the effectiveness of this technique improves with increasing frequency up to frequencies exceeding $\omega_{\text{NV}}$ by almost an order of magnitude, engendering optimism that this approach can be extended to higher frequencies, paving the way to probe spin dynamics in a wide variety of AFMs using QSSs. Last, we exploit the spatial localization of these field fluctuations to a length scale set by the magnon wavelength, which, in tandem with the highly localized sensitivity of the nano-scale QSS, reveals magnon transport over length scales of tens of micrometers.

## RESULTS

### Materials studied

**Fig. 1.**
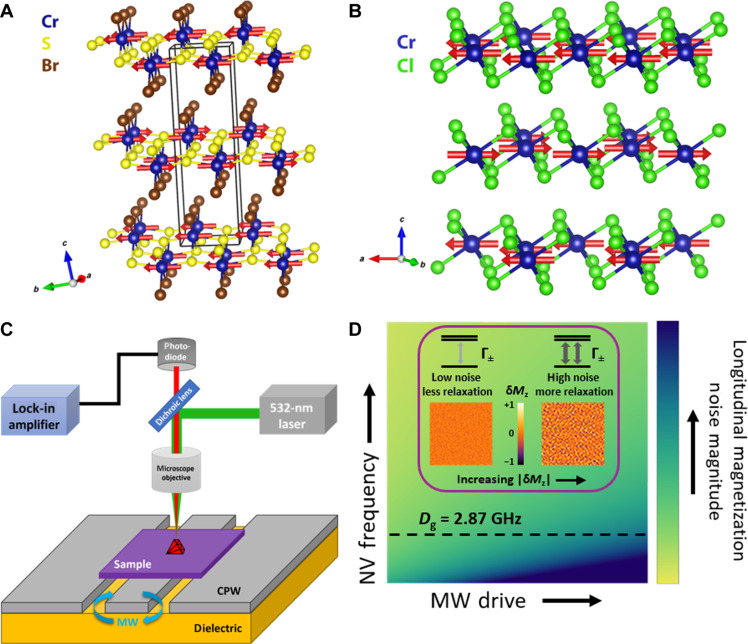
Overview of optical detection of high-frequency magnetic dynamics in vdW AFMs enabled by magnetic field noise arising from diffusing magnons. (**A** and **B**) Schematic side view of the crystal and spin structures of CrSBr (A) and CrCl_3_ (B), with spins indicated by red arrows. (**C**) Schematic of the setup for optical detection of antiferromagnetic resonance. A sample flake is exfoliated and transferred onto a microwave coplanar waveguide. Nanodiamonds containing NV^−^ centers are dropcast onto the sample surface, and photoluminescence is measured under continuous laser excitation as a function of microwave frequency and applied in-plane magnetic field. (**D**) Schematically depicts the magnetic noise magnitude as a function of the microwave drive power (horizontal axis) and the QSS frequency (vertical axis). Driving AFMR increases the magnon population in the sample, in turn increasing low-frequency magnetic noise even when the single-magnon gap is much larger than the QSS frequency as described in the main text and the Supplementary Materials. The inset in (D) depicts the enhancement of the QSS relaxation rate due to the collective diffusive dynamics of the magnon gas at larger magnon density.

**Fig. 2.**
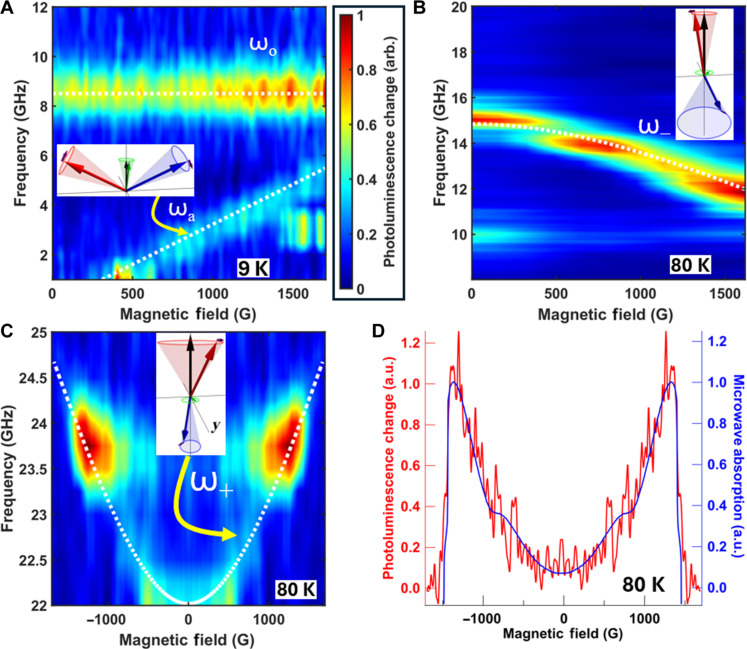
Optically detected antiferromagnetic resonance spectra up to 24 GHz. (**A**) Optically detected CrCl_3_ AFMR dispersion showing the optical mode $\omega_{o}$ and acoustic mode $\omega_{a}$ ; the white dashed lines show fits using Eq. 4. The insets in (A) to (C) depict the motion of the AFM sublattice magnetizations in spin space in response to AFMR excitation. Sublattice magnetizations $M_{A}$ and $M_{B}$ are shown as red and blue arrows, respectively, the net magnetization in green, and applied field in black. The cones trace out the motion of the magnetization over a cycle, with the solid ellipses tracing the path of the arrowhead. The small purple arrows indicate the direction of precession of each sublattice. The equilibrium orientations about which the sublattice magnetizations precess are shown in fig. S4 (A and B). (**B** to **D**) Optically measured dependencies of the CrSBr AFMR mode frequencies on magnetic field: (B) shows the low-frequency mode $\omega_{-}$ [see Eq. 5 for $\omega_{-}\left(H\right)$ and $\omega_{+}\left(H\right)$ ], while (C) shows the high-frequency mode $\omega_{+}$ . The internal fields corresponding to the white dashed lines are presented in Fig. 3D. (D) Linecuts through the data in (C) at 23.75 GHz comparing the optically detected CrSBr $\omega_{+}$ AFMR mode (red) with simultaneously measured inductive AFMR (blue). a.u., arbitrary units.

We studied two vdW AFMs: CrCl_3_ and CrSBr (whose Néel temperatures $T_{N}$ are 14 and 132 K, respectively) with dimensions approximately 1 mm–by–1 mm–by–50 μm thick. These collinear AFMs consist of two oppositely oriented but equally magnetized spin sublattices that can be viewed as interleaved ferromagnetic sublattices [see Fig. 1 (A and B)]. Static stray fields vanish except at inhomogeneities such as grain boundaries, step edges, or domain walls. However, finite temperature and microwave (MW) magnetic field drive of the uniform AFMR mode excite magnons, causing sublattice precession that creates a small net magnetization due to canting (see insets in Fig. 2) (*18*, *19*) and hence stray fields that extend above the surface over distances set by the inverse of the magnon wavevector *k* (*20*).

The samples were placed on a copper coplanar waveguide (CPW), and nanodiamonds containing NV^−^ centers were dropcast onto the top surface as schematically depicted in Fig. 1C. A static magnetic field is applied parallel to the sample plane, and the CPW generates microwave magnetic fields. The CrCl_3_ ODAFMR was measured with the static magnetic field applied in-plane but perpendicular to the CPW center conductor in a parallel-pumping geometry. Given the small in-plane anisotropy of CrCl_3_, the sublattices maintain a spin-flop configuration resulting in optical and acoustic AFMR modes (*7*). CrSBr exhibits a larger in-plane anisotropy, that is, its anisotropy is biaxial (*8*); the CrSBr was oriented with the easy-anisotropy (*b*) axis oriented parallel to the applied field (see fig. S4 and related discussion in the Supplementary Materials for discussion of magnetic configurations). The excitation of the uniform AFMR mode thus excites modes that are hybrids of the right-handed and left-handed uniaxial AFMR modes (*8*). The insets in Fig. 2 depict these modes in spin space. We measure the magnetic field dependence of the change in photoluminescence in response to excitation of AFMR at various microwave frequencies using a lock-in amplifier while modulating the amplitude of the applied microwave field (Fig.2).

### Principles of ODAFMR

The relative occupation of the NV^−^ spin states can be manipulated by microwave magnetic fields matching the ESR frequency. Coherent irradiation resonantly drives Δm=±1 transitions between NV^−^ spin states (ESR), while stochastic fluctuating fields relax a spin system that is out of thermal equilibrium with the thermal reservoir to which it is coupled by these fluctuating fields—the magnon gas in this case—as is the case for NV^−^ spins when they are hyperpolarized by laser irradiation. The magnetic Hamiltonian of the NV^−^ spin in an applied field B is given by (*21*)

$$\begin{array}{c} \frac{{\hat{ℋ}}_{\text{NV}}}{h}=D_{g}\left[{\hat{S}}_{z}^{2}-\frac{S\left(S+1\right)}{3}\right]+E_{g}\left({\hat{S}}_{x}^{2}-{\hat{S}}_{y}^{2}\right)+\frac{\tilde{\gamma }}{2\pi }B\cdot \hat{S} \end{array}$$ (1)

where h is the Planck’s constant, $\tilde{\gamma }/2\pi =2.8$ MHz/G is the electron gyromagnetic ratio, $D_{g}=2.87$ GHz is the ground-state zero-field splitting, $E_{g}$ is the off-axis splitting, S=1 is the NV^−^ spin magnitude, and $\hat{S}={\left[{\hat{S}}_{x},{\hat{S}}_{y},{\hat{S}}_{z}\right]}^{T}$ is the vector of spin 1 Pauli matrices. Thus, stochastic electromagnetic fluctuations matching the $m_{s}=0\;↔\;m_{s}=\pm 1$ transition frequencies will relax the NV^−^ spin polarization, enhancing the population of the less radiative $m_{s}=\pm 1$ states and reducing photoluminescence. This is the principle of NV^−^ center relaxometry as a method for optically detecting magnetic dynamics arising from the excitations of AFMR.

The fluctuating magnetic fields that relax the NV^−^ spin result from the diffusive collective dynamics of the magnon gas in the proximate vdW magnet. Thus, even when the single-magnon AFMR dispersion has a large spectral gap and hence does not contribute directly to magnetic noise at the NV^−^ ESR frequency $\omega_{\text{NV}}$ , magnon-magnon processes generate electromagnetic fluctuations whose spectrum extends to very low frequency (*14*, *22*–*25*) as schematically illustrated in Fig. 1D. In the absence of microwave drive, the density of magnons is described by a thermal magnon population. When the uniform ( k=0 ) AFMR mode is resonantly excited by monochromatic microwaves, the resultant enhanced spin-density decays into finite-momentum ( k≠0 ) magnons through nonlinear interactions in the Hamiltonian (see the Supplementary Materials for details). This increased population of magnons subsequently generates strong low-frequency magnetic field fluctuations that match the NV^−^ spin-transition frequency, thereby enhancing the NV^−^ relaxation rate.

To formalize the above intuition, we note that the NV^−^ relaxation rate resulting from stochastic field fluctuations is given by the spectral density Γ(ω) of these stray magnetic fields ${\hat{B}}^{\pm }$ evaluated at $\omega_{\text{NV}}$

$$\Gamma \left(\omega_{\mathrm{NV}}\right)=\frac{\gamma^{2}}{2}\int \langle {\hat{B}}^{+}\left(0\right){\hat{B}}^{-}\left(t\right)\rangle e^{-i\omega_{\mathrm{NV}}t}dt$$ (2)

where γ is the NV^−^ gyromagnetic ratio. The larger number of finite wave vector magnons that are excited indirectly via microwave driving of the uniform AFMR mode enhances the amplitudes of the stray fields $\hat{B}\left(t\right)$ and hence $\Gamma \left(\omega_{\text{NV}}\right)$ . More explicitly, the stray field correlation function can be expressed in terms of the longitudinal spin fluctuations $C_{∥}\left(k,\omega_{\text{NV}}\right)$ of the magnon gas, as

$$\Gamma \left(\omega_{\mathrm{NV}}\right)\propto \int d^{2}{\mathrm{kF}}_{d}\left(k\right)C_{∥}\left(k,\omega_{\mathrm{NV}}\right)$$ (3)

where $F_{d}\left(k\right)=k^{2}e^{-2\mathrm{kd}}$ is a momentum filter function that describes the propagation of the stray field fluctuations from the source (magnet) to the sensor (NV^−^) which is at a distance *d*. (*22*, *25*, *26*). Assuming that the magnon-density *n* is approximately conserved in the steady state, the magnetization density is also conserved, and the longitudinal spin-correlation function $C_{∥}\left(k,\omega_{\text{NV}}\right)$ takes a diffusive form, with a diffusion constant $D_{s}$ that scales inversely with *n*. In this limit, $\Gamma \left(\omega_{\text{NV}}\right)\propto D_{s}^{-2}\propto n^{2}$ (see the Supplementary Materials for a derivation), implying that the NV^−^ relaxation rate is enhanced due to a larger density of magnons. Consequently, the uniform AFMR mode may be characterized directly via NV^−^ relaxometry through microwave driving, despite its spectral gap being much larger than $\omega_{\text{NV}}$ , as we show below.

### ODAFMR measurements

We demonstrate the efficacy of optically detected AFMR spectroscopy using NV^−^ relaxometry in two widely studied vdW AFMs: CrCl_3_ and CrSBr. For this emergent approach, we first confirm in both cases that spectra obtained are in agreement with conventionally obtained microwave measurements and enable spectroscopic determination of key magnetic parameters such as exchange interactions and anisotropies. ODAFMR spectra, that is, the reduction of the photoluminescence (PL) as a function of microwave frequency and static in-plane magnetic field, measured in CrCl_3_ at 9 K are shown. We then study CrSBr at five temperatures cooling from 120 K—where AFMR vanishes at a transition to what we believe is a spin-flop phase—down to 80 K where the AFMR frequency rises to 24 GHz.

We observe two CrCl_3_ AFMR modes (see Fig. 2A) corresponding to the acoustic (lower) and optical (upper) AFMR modes, at frequencies $\omega_{a}$ and $\omega_{o}$ , respectively (*7*)

$$\begin{array}{c} \omega_{a}\left(H\right)=\mu_{0}\gamma \sqrt{\frac{2H_{E}+M_{S}}{2H_{E}}}H\;\text{and}\;\omega_{o}\left(H\right)=\mu_{0}\gamma \sqrt{2H_{E}M_{S}\left(1-\frac{H^{2}}{4H_{E}^{2}}\right)} \end{array}$$ (4)

where $H_{E}$ is the exchange field, $M_{S}$ is the saturation magnetization, and γ is the gyromagnetic ratio of CrCl_3_. In the field range accessible to our setup, the optical mode $\omega_{o}$ appears approximately constant at about ~8.5 GHz. Consistent with previous AFMR measurements obtained using conventional microwave absorption techniques (*7*), this mode is relatively broad in frequency. Fitting our optical data yields estimates of the exchange field and saturation magnetization of CrCl_3_: $\mu_{0}H_{E}=\left(2.64\pm 0.17\right)$ kG and $\mu_{0}M_{S}=\left(1.75\pm 0.11\right)$ kG at 9 K assuming γ/2π=2.8 MHz/G. Simultaneous inductive AFMR measurements (see fig. S2A and the Supplementary Materials), manifesting as a reduction in the microwave transmission $S_{12}$ , give similar values of $\mu_{0}H_{E}=\left(2.58\pm 0.22\right)$ kG and $\mu_{0}M_{S}=\left(1.78\pm 0.15\right)$ kG.

**Fig. 3.**
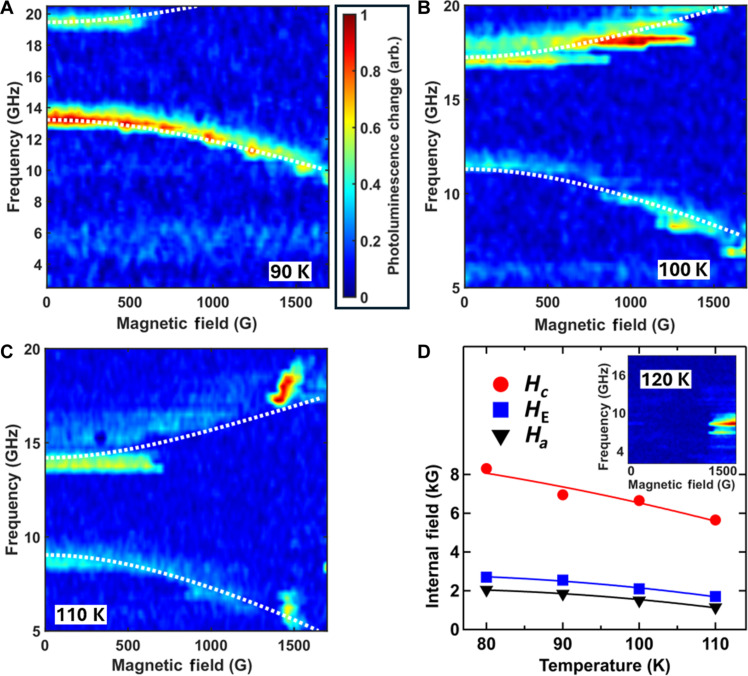
Determination of internal fields in CrSBr from optically detected AFMR spectra. (**A** to **C**) Temperature evolution of the CrSBr dispersions of the lower-frequency $\omega_{-}$ and the higher-frequency $\omega_{+}$ modes both of which decrease with increasing temperature: (A) shows 90 K, and (B) shows 100 K. In (C), taken at 110 K, both modes are visible along with a feature at 1500 G that corresponds to either the spin-flop transition or a field-induced ferromagnetic mode. (**D**) Temperature dependencies of the internal fields $H_{c}$ , $H_{E}$ , and $H_{a}$ ; the white dashed curves in Fig. 2 (B and C) and in (A) to (C) show the mode dispersions predicted by these internal fields through Eq. 5. Inset in (D): No AFMR modes are observed at 120 K, but the feature near 1500 G intensifies.

**Fig. 4.**
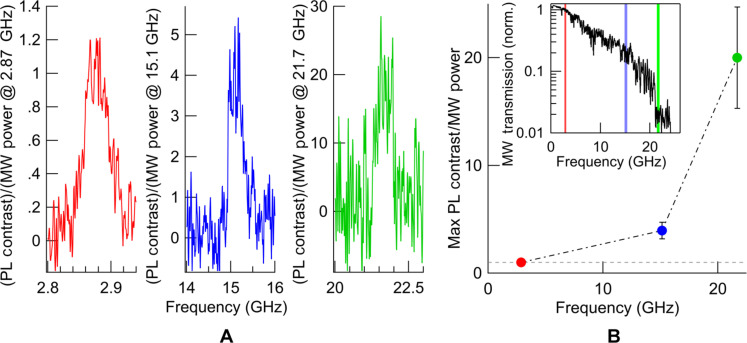
Strength of optically detected magnetic resonance signals when normalized by microwave power. (**A**) Photoluminescence contrast signals plotted on three different vertical scales to account for the substantial decrease of available microwave power at higher frequencies. We compare the directly excited ESR (red), normalized to unity, with the 15.1-GHz (blue) and 21.7-GHz (green) CrSBr AFMR modes at 80 K. The available microwave power, shown as an inset in (B) with vertical dashed lines at these frequencies, decreases by more than an order of magnitude between 15.1 and 21.7 GHz. (**B**) Frequency dependence of the size of the normalized signals obtained from the plots in (A). Correcting for this reduced power, the sizes of the signals of the low- and high-frequency modes exceed the direct NV^−^ ESR by factors of 4.5 and 20, respectively.

Turning to CrSBr, another easy-plane AFM with hard and soft magnetic axes in the easy plane, we present ODAFMR spectra obtained over a wider range of temperatures to demonstrate the effectiveness of ODAFMR as a local magnetic spectroscopy tool to determine magnetic parameters; these are shown in Fig. 3. Given the biaxial anisotropy in CrSBr (the hard *c* axis is perpendicular to the intermediate *a* axis), the AFMR modes $\omega_{\pm }$ with H parallel to the easy *b* axis are hybridizations of the uniaxial right-handed and left-handed modes (*8*, *19*)

$$\begin{array}{c} \begin{array}{c} \frac{\omega_{\pm }\left(H\right)}{\mu_{0}\gamma }=\\ \sqrt{H^{2}+H_{a}\left(H_{E}+H_{c}\right)+H_{E}H_{c}\pm \sqrt{H^{2}\left(H_{a}+H_{c}\right)\left(H_{a}+4H_{E}+H_{c}\right)+H_{E}^{2}{\left(H_{a}-H_{c}\right)}^{2}}} \end{array} \end{array}$$ (5)

where the subscript ± refers to higher and lower frequencies and are unrelated to the superscripts used in Eq. 2. Here, $H_{c}$ and $H_{a}$ are the hard and intermediate axis anisotropies, respectively. Figure 2B shows ODAFMR of the lower mode $\omega_{-}$ at 80 K. To enhance the magnon density, data were typically taken at input microwave power of 30 dBm, a power sufficient that an asymmetric line shape characteristic of nonlinear response (*23*) is observed (see the Supplementary Materials). However, transmission losses substantially reduce the power delivered to the CPW: At the highest frequencies, the power applied to the CPW is reduced by approximately two orders of magnitude (see the inset in Fig. 4B). The microwave drive and the laser increase the sample temperature above ambient by less than 5 K (see the Supplementary Materials).

The CrSBr ODAFMR spectra obtained at 90, 100, 110, and 120 K are shown in Fig. 3. Values of the effective fields obtained from the optical data are shown in Fig. 3D: the effective fields $H_{E}$ , $H_{c}$ , and $H_{a}$ and hence both modes $\omega_{\pm }$ decrease with increasing temperature as previously observed (*8*). At 120 K, a feature arising from either a spin-flop or ferromagnetic mode appears as temperature approaches $T_{N}$ . Similar features are also observable around 1500 G at 110 K.

## DISCUSSION

On the basis of our results, we discuss two salient observations that highlight the efficacy of optically detected relaxometry for understanding spin dynamics in AFMs. First, we show that the local sensitivity of the QSS can be leveraged to demonstrate transport of antiferromagnetic magnons across the 50-μm thickness of the CrSBr crystal, revealing long-range magnon transport indicative of a magnon mean–free path comparable to the crystal thickness (tens of micrometers). Our observation is consistent with recent magneto-optical observations of long-ranged spin transport in CrSBr (*27*, *28*). Second, we demonstrate relaxometric detection of ODAFMR detection extending to 24 GHz, the highest frequency reported to date and eight times the QSS resonance frequency. We further find that, at constant CPW-generated microwave magnetic field intensity, the efficiency of ODAFMR detection substantially increases with increasing frequency, thereby enabling broadband detection of spin dynamics.

To show long-range magnon transport, we begin by noting that the sensitivity of the QSS to the fluctuating stray magnetic fields emanated by the magnon gas in the CrSBr sample is highly local. Because of the evanescent nature of the magnon-generated electromagnetic wave, the sensitivity of the QSS to the collective magnetic mode decreases exponentially with the mode wave vector *k* as described by the filter function $F_{d}\left(k\right)=k^{2}e^{-2\mathrm{kd}}$ (*14*, *22*, *26*) mentioned earlier, where *d* is the separation of the QSS source of the stray field. Consequently, this suppressed coupling to magnetic modes at the bottom of the sample (50-μm thick) minimizes the contribution of magnons located there to QSS relaxation.

At the same time, the current density in the CPW center conductor is confined to its edges due to a combination of the skin effect (skin depth $\delta_{s}$ of ~1 μm at 10 GHz) and charge bunching (also known as the proximity effect) (*29*, *30*). This edge confinement of current filaments attenuates the microwave fields at the top of the sample 50 μm above the CPW. This decreased microwave field strength is confirmed by our NV ESR measurements that revealed ESR signals much weaker than those from NV^−^ centers in nanodiamonds deposited on the CPW. These microwave fields are weaker still at AFMR frequencies (relative to microwave excitation power—see below): too weak to excite AFMR at the top of the sample sufficiently to account for the rapid QSS relaxation rates we observe. This implies that magnons generated at the bottom of the crystal readily diffuse tens of micrometers to the top of the sample where they couple strongly to the QSS, thus enhancing its relaxation rate.

To quantify the above observation, we compare the strength of the coherently driven ESR signal to the relaxometric signal (remembering that, in both cases, the fluctuating fields match the QSS resonance frequency, but the fields that relax the QSS originate from excitation of AFMR at much higher frequencies). Because these signals grow linearly with microwave power, we compare the strength of ESR—a measure of the square of the microwave fields generated by the CPW 50 μm below—versus AFMR signals—a measure of magnon-generated fields—normalized by the microwave power delivered to the CPW at the relevant drive frequencies (Fig. 4A). Because of losses in the CPW and associated transmission lines, the power delivered at the higher AFMR frequencies (up to 24 GHz) is reduced by up to two orders of magnitude (see the inset in Fig. 4B). Nevertheless, a comparison of power-normalized signals in Fig. 4 shows that the signal generated by exciting the higher-frequency (21.7 GHz) AFMR mode actually exceeds the directly driven NV^−^ ESR by over an order of magnitude. This demonstrates remarkably strong fluctuating fields arising from a high density of finite wave vector magnons at the top surface of the thick crystal. The CPW-generated microwave magnetic fields at AFMR frequencies are even weaker at the top surface than at ESR frequencies due to the frequency dependence of charge bunching. This further reduces the ability of the distant CPW to drive AFMR sufficiently to generate magnon densities required for this strong relaxometric signal. Instead, they must arise from magnons generated adjacent to the CPW that then diffuse to the top surface of the crystal and accumulate there. This observation of magnon transport over tens of micrometers to the NV^−^ detectors atop the crystal enhances the promise of antiferromagnetic magnonics for spintronic applications.

Our second observation emphasizes the potential relaxometry holds for application of spatially localized AFMR to higher frequencies still: The effectiveness of this relaxometric detection of AFMR by QSS increases with increasing frequency up to the highest frequencies we measured. Figure 4B shows this frequency dependence: The power-normalized signal at 21.7 GHz exceeds that at 15.1 GHz by a factor of 4.4. Future studies will seek to clarify the mechanism for this.

In summary, we show that QSSs are effective local optical probes of antiferromagnetic dynamics in the vdW AFMs CrCl_3_ and CrSBr. Specifically, we characterize magnetic exchange fields and anisotropies using optically detected AFMR and illuminate underlying magnetization dynamics and excitations. The localized sensitivity of the sensors to fluctuating stray fields generated by the antiferromagnetic magnons reveals magnon transport over tens of micrometers in these materials. Studies of CrSBr were performed at frequencies up to 24 GHz, eight times the NV^−^ ESR frequency, and AFMR detection efficiency was found to increase with AFMR frequency. By exploiting nonequilibrium magnon dynamics to infer high-frequency spectroscopic features of AFMs, our work adds a dimension to existing and concurrent studies of equilibrium low-energy spin dynamics in two-dimensional (2D) magnets using spin sensors (*24*, *31*–*35*). Further, our observations of long-range magnon transport indicate the potential for 2D AFMs to excel in high-frequency spintronic applications.

Magnetic resonance is a proven tool for spectroscopic characterization of interactions and dynamics in magnets. While conventional inductively detected magnetic resonance techniques (microwave power absorption) lack the sensitivity and spatial localization needed for spatially localized studies, this work demonstrates that QSS-based ODAFMR overcomes this barrier to local studies of AFMs. Another promising avenue for expanding the scope of this technique lies in the application of the boron vacancy center embedded in hexagonal boron nitride (*36*), whose spin-dependent optical properties are similar to those of the NV^−^ center (*37*), but offers the advantage that it can be seamlessly integrated with a variety of 2D magnetic systems.

## MATERIALS AND METHODS

### Materials

CrCl_3_ crystals (fig. S1A) obtained from J. Goldberger were grown using a typical chemical vapor transport method. Anhydrous CrCl_3_ (99.99%, Alfa Aesar) was weighed out in an Ar-filled glovebox and sealed in an evacuated quartz ampoule (270-mm length, 15-mm inner diameter, and 1-mm wall thickness). The ampoule was heated in a three-zone furnace for 18 to 72 hours. The feed zone, which contained the reactant mixture, was kept at 650° to 700°C, while the crystals grew in the growth zone at 500° to 550°C. The resulting crystalline platelets had a purple color. Purity was confirmed by powder x-ray diffraction. The high-quality CrSBr single crystals (fig. S1B) obtained from B. Lv were synthesized through a direct solid-vapor reaction in a box furnace, as described in detail in (*10*). Both samples were exfoliated to a thickness of ~50 μm.

### Experimental configuration

Experiments were performed using a custom CPW with a center conductor width of 330 μm, a thickness of 35 μm, and with the dielectric FR-4 from Advanced Circuits. The samples were placed on the center line of the microwave CPW. Nanodiamonds containing NV^−^ centers were then dropcast onto the surfaces of the samples, resulting in a nonuniform distribution. Room temperature optically detected NV^−^ ESR verified their presence on the surface of the CrCl_3_ (fig. S1C) and CrSBr (fig. S1D). As expected, no ODAFMR signal is visible for either sample at room temperature.

### Antiferromagnetic resonance measurements

Antiferromagnetic resonance measurements are performed in vacuum at cryogenic temperatures in an optical cryostat. The cryostat is positioned such that the sample space lies at the center of an electromagnet whose magnetic field lies in the plane of both the sample and the coplanar waveguide. The applied field was parallel to the MW field for all CrCl_3_ measurements, and the applied field was perpendicular to the MW field for all CrSBr data [see fig. S1 (A and B)].

The primary independent variables in these measurements are the magnetic field strength and direction, the microwave power and frequency, and the temperature. Microwave frequency electric current is sent through the coplanar waveguide using a microwave generator. The microwave amplitude is modulated at low frequency to allow lock-in detection of the signal. The transmitted microwave amplitude is converted into a modulated dc voltage using a microwave diode, and the lock-in detects this voltage using the modulation frequency as the reference frequency. At its resonance field and frequency, AFMR is inductively measured as a reduction of the transmitted microwave power.

Simultaneously, green (532 nm) light generated by a laser is continuously applied to nanodiamonds on the sample surface containing NV^−^ centers. The red photoluminescence (with green light filtered out passing >637 nm) from the NV^−^ centers is continuously collected by a photodiode whose output current is fed to a current-to-voltage preamplifier and then fed into a second lock-in amplifier referenced to the same modulation frequency. AFMR is optically measured as a reduction of emitted photoluminescence of the NV^−^ centers.

## Supplementary Materials

### This PDF file includes:

Figs. S1 to S4 Supplementary Text References
