# Supplementary material for: Quantum sensing of broadband spin dynamics and magnon transport in antiferromagnets
Source: Sci Adv. 2025 Jun 27;11(26):eadu9381. doi: 10.1126/sciadv.adu9381 (PMC12204117; doi:10.1126/sciadv.adu9381)
Supplement: Supplementary file 1 — Figs. S1 to S4 Supplementary Text References [file sciadv.adu9381_sm.pdf]

Science Advances
AAAS

Supplementary Materials for

Quantum sensing of broadband spin dynamics and magnon transport in antiferromagnets

Alex L. Melendez *et al.*

Corresponding author: Simranjeet Singh, simranjs@andrew.cmu.edu; P. Chris Hammel, hammel.7@osu.edu

Sci. Adv. **11**, eadu9381 (2025)
DOI: 10.1126/sciadv.adu9381

This PDF file includes:

Figs. S1 to S4
Supplementary Text
References

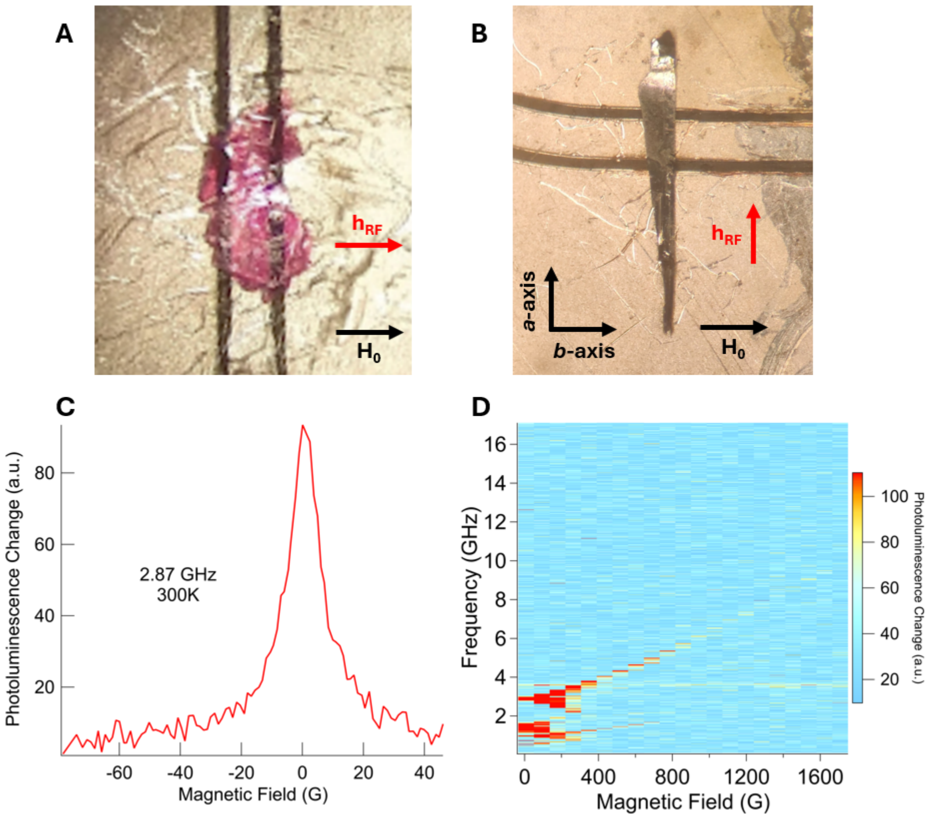

Figure S1: **Arrangement of samples with associated ESR spectra.** Optical images of $CrCl_3$, panel (A), and CrSBr, panel (B), on a microwave coplanar waveguide. The magnetic field $\boldsymbol{H}_0$ is applied along the left-right axis for both samples. CrSBr is oriented such that the easy-axis and the Néel vector (crystallographic b-axis) are aligned along $\boldsymbol{H}_0$. Panel (C): Optically detected electron spin resonance (ODESR) of nanodiamonds deposited on $CrCl_3$ surface at room temperature at 2.87 GHz. Panel (D): ODESR of nanodiamonds on CrSBr surface at room temperature. The data shown in panels (C) and (D) were obtained using a laser power of 5 mW.

Aligning applied field with CrSBr easy-axis

The easy anisotropy axis of the CrSBr was determined via a combination of visual cues and the measured AFMR dispersion. When bulk CrSBr is viewed under an optical microscope and exfoliated, the difference between the two in-plane crystal axes (a-axis and b-axis) are visually evident due to the clear difference in their proclivity to cleave along the planes perpendicular to these directions. In particular, CrSBr tends to cleave along the plane perpendicular to the to the

b-axis much more easily than cleaving along the plane perpendicular to the to the *a*-axis. We confirm this with measurements of the AFMR signal as a function of applied field and frequency in both geometries, that is, with the magnetic field parallel to and perpendicular to the *b*-axis. The dispersions obtained were consistent with the *b*-axis being the easy anisotropy axis and the *a*-axis being the intermediate anisotropy axis. This enabled us to align the applied magnetic field and the CPW center conductor such that both are parallel to the CrSBr easy-axis.

Inductively Detected AFMR in $CrCl_3$

Simultaneously with NV^- photoluminescence measurements, AFMR was inductively measured by monitoring the reduction of microwave transmission (S_{12}) at 9K on $CrCl_3$, as shown in Fig. S2A. The frequency dependence of MW transmission between 0 and 12 GHz required normalization of the inductive signal using MW transmission values obtained away from the resonance condition. The $CrCl_3$ optical and inductive signals at 9K and 4 GHz are compared in Fig. S2B.

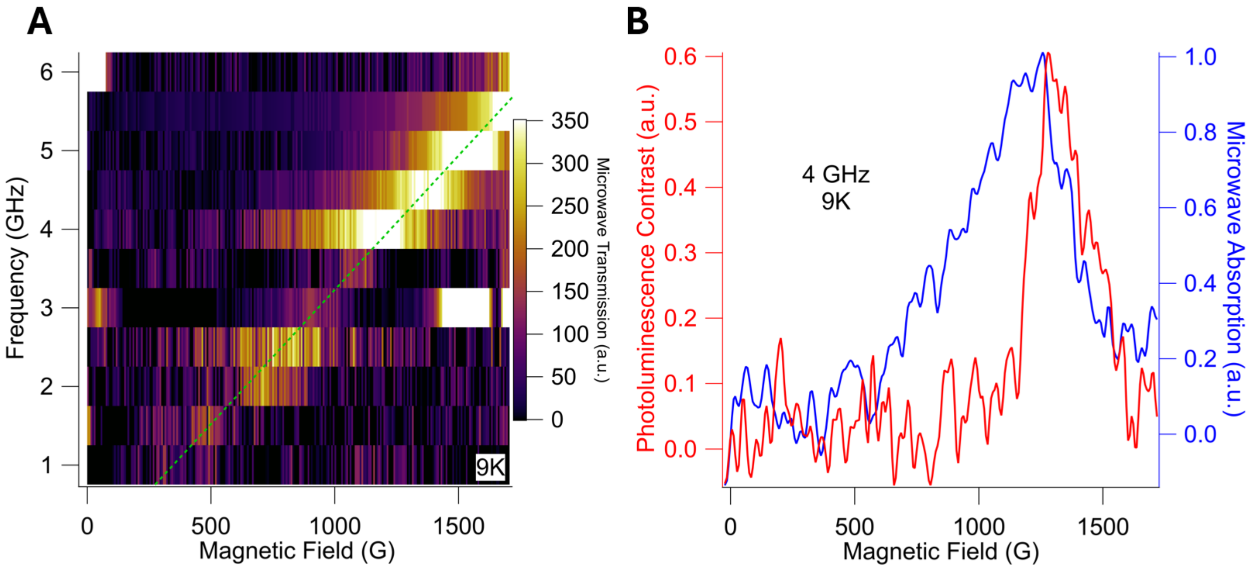

Figure S2: **Comparison of inductive and optically detected AFMR spectra.** Panel (A): Inductive AFMR (MW absorption) signal corresponding to the $CrCl_3$ optical data at 9K shown in Fig. 2A of the main paper. Dotted line is a fit to the MW absorption peaks as described in the main text following Eq. 4 for the acoustic AFMR mode. Panel (B) compares optically detected [red] and inductively detected [blue] AFMR signals from $CrCl_3$ at 4 GHz and 9 K.

Dependence of AFMR on Laser Power

We measured the heating of the CrSBr sample by the laser (wavelength 532 nm) for the powers up to 5 mW used in our measurements. This done by comparing to inductive AFMR measurements performed at low (-10 dBm) MW power were performed in the presence of laser illumination at powers 0, 1mW and 5mW (Fig. S3). To calibrate the temperature change associated with the change of the resonance field, the temperature dependence of the resonance field at 11 GHz was taken from 90 to 107.5 K which revealed a reduction of the resonance field of (76.4 ± 4.7) G/K. Together with the change in the resonance field shown in Fig. S3 this yields an estimated temperature increase of (0.8 ± 0.5) K at 1 mW and (3.3 ± 0.5) K at 5 mW.

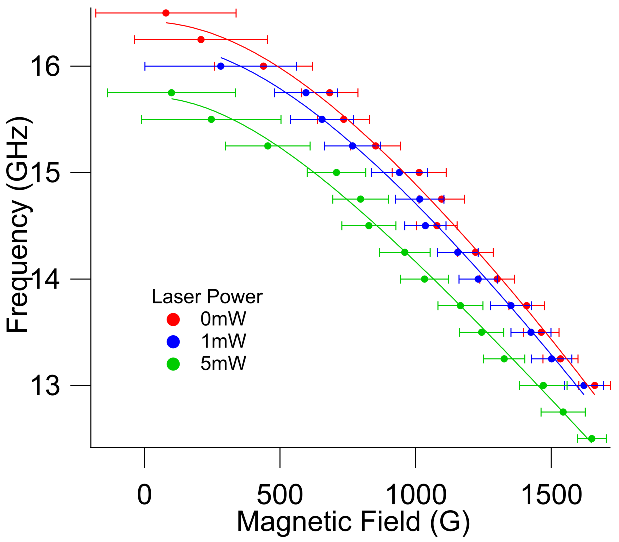

Figure S3: **Dependence of resonance frequencies on applied laser power in CrSBR.** Inductive AFMR signal of CrSBr low frequency mode at 80 K, measured at three different laser powers. Solid curves show fits to Eq. (5), main text. Error bars represent FWHM of Lorentzian fits.

Magnon-Scattering in CrSBr and Enhancement of NV^- Relaxation Rate

Here we work out the non-linear spin-wave Hamiltonian for CrSBr, and show that the quadratic terms lead to the magnon dispersions used in the main text, while the higher order terms lead to magnon-scattering. Next, we argue that when the zero-momentum magnon mode (AFMR mode) is pumped via resonant micro-wave excitation, it will decay via magnon-scattering into finite momentum

modes, leading to spin transport. Finally, we show that the increased density of AFMR mode magnons will decrease the spin diffusion constant D_s (assuming approximate number conservation in the steady state), leading to an increase in the NV^- relaxation rate.

The Hamiltonian for CrSBr is given by an in-plane ferro-magnetic coupling and inter-plane anti-ferromagnetic coupling between Cr^{3+} spin $\boldsymbol{S}_{n,i}$ (where n is the plane index and i is the position of the spin in-plane), with a biaxial anisotropy that prefers alignment along the b axis in the ab-plane, with the a axis being the intermediate axis and the c axis being the hard axis.

$$\mathcal{H} = -\sum_{n,i,\delta} J_\delta \boldsymbol{S}_{n,i} \cdot \boldsymbol{S}_{n,i+\delta} + J_E \sum_{n,i} \boldsymbol{S}_{n,i} \cdot \boldsymbol{S}_{n+1,i} + \frac{D_c}{2} \sum_{n,i} \left(S^c_{n,i}\right)^2 + \frac{D_a}{2} \sum_{n,i} \left(S^a_{n,i}\right)^2 - g\mu_B \sum_{n,i} \boldsymbol{B} \cdot \boldsymbol{S}_{n,i} \tag{S1}$$

where J_δ refer to the in-plane coupling between spins at sites connected by $\boldsymbol{\delta}$ —the most significant ones are $J_2 = 3.88$ meV for $\boldsymbol{\delta} = (\pm a\hat{a} \pm b\hat{b})/2$, $J_1 = 2.79$ meV for $\boldsymbol{\delta} = \pm a\hat{a}$, and $J_3 = 2.12$ for $\boldsymbol{\delta} = \pm b\hat{b}$ from first principles calculations in Ref. (*38*); slightly smaller values are obtained from neutron-scattering experiments in Ref. (*39*). The anisotropies D_a and D_c and the inter-plane coupling J_E are expected to be much smaller, of the order of 10-100 μeV. They are related to the effective fields referenced in the main text by $H_{a(c)} = -D_{a(c)}S/g\mu_B$ (similarly $H_E = -J_E S/g\mu_B$), where g is the Landé g-factor, μ_B is the Bohr magneton and S is the magnitude of the spin. The small anisotropy terms $D_{a/c}$ break the continuous spin-rotation symmetry about the ordering direction $\hat{b}$ to discrete spin-rotation symmetries in H, indicating that the longitudinal spin-density will only be approximately (but not exactly) conserved by the system's dynamics.

The magnon dispersion for CrSBr has been derived using a Landau-Lifshitz approach (*8*), but it does not readily provide the magnon-scattering terms. Therefore, to simultaneously derive both, we use non-linear spin-wave theory, *i.e.*, we write the spin operator in terms of Holstein-Primakoff bosons and carry out a 1/S expansion (*40*). For simplicity, we consider the case of a bilayer with $n = 1, 2$, which is sufficient to capture the physics of interest. In the first layer, the spins are aligned along the easy axis, *i.e.*, $\langle \boldsymbol{S}_{1,i} \rangle = S\,\hat{b}$, while in the next layer, the spins are anti-aligned with the first layer, such that $\langle \boldsymbol{S}_{2,i} \rangle = -S\,\hat{b}$, with $S = 3/2$. We also consider $\boldsymbol{B} = B\hat{b}$ along the easy-plane, as in the experiment, and remain in the regime where B is small enough to avoid a spin-flop transition. Next, we define the spin-operators in terms of Holstein-Primakoff bosons a and b for the top and

bottom layers as ($\hbar = 1$)

$$S^b_{1,i} = S - a_i^\dagger a_i,\ S^+_{1,i} = S^c_{1,i} + iS^a_{1,i} = (2S - a_i^\dagger a_i)^{1/2} a_i,\ S^-_{1,i} = (S^+_{1,i})^\dagger = a_i^\dagger (2S - a_i^\dagger a_i)^{1/2},$$
$$S^b_{2,i} = -S + b_i^\dagger b_i,\ S^+_{2,i} = S^c_{2,i} + iS^a_{2,i} = b_i^\dagger (2S - b_i^\dagger b_i)^{1/2},\ S^-_{1,i} = (S^+_{1,i})^\dagger = (2S - b_i^\dagger b_i)^{1/2} b_i \quad \text{(S2)}$$

Expanding the Hamiltonian and carrying out an expansion order by order in 1/S, we arrive at the following Hamiltonian (N = total number of sites in plane):

$$\begin{aligned}
\mathcal{H} &= \mathcal{H}^{(0)} + \mathcal{H}^{(2)} + \mathcal{H}^{(4)} + \dots \\
\mathcal{H}^{(0)} &= -2NS^2 \left(\sum_\delta J_\delta \right) - J_E N S^2 \\
\mathcal{H}^{(2)} &= S \Bigg[\sum_{i,\delta} J_\delta \left(a_i^\dagger a_i + a_{i+\delta}^\dagger a_{i+\delta} - a_i^\dagger a_{i+\delta} - a_{i+\delta}^\dagger a_i \right) + \sum_i \frac{(D_a - D_c)}{4} [a_i^2 - (a_i^\dagger)^2] \\
&\quad + \frac{(D_c + D_a)}{4} (a_i^\dagger a_i + a_i a_i^\dagger) \Bigg] + (a_i, \to b_i, a_i^\dagger \to b_i^\dagger) + g\mu_B B \sum_i (a_i^\dagger a_i - b_i^\dagger b_i) \\
&\quad + J_E S \left[a_i^\dagger a_i + b_i^\dagger b_i + a_i^\dagger b_i^\dagger + a_i b_i \right] \\
\mathcal{H}^{(4)} &= \frac{1}{4} \sum_{i,\delta} J_\delta \left[a_i^\dagger a_{i+\delta} a_{i+\delta}^\dagger a_i - a_i^\dagger (a_i)^2 a_{i+\delta}^\dagger - a_i (a_{i+\delta}^\dagger)^2 a_{i+\delta} + \text{H.c.} \right] + \frac{1}{8} \sum_i (D_a - D_c) [a_i a_i^\dagger (a_i)^2 + (a_i^\dagger)^3 a_i] \\
&\quad + \frac{1}{8} \sum_i (D_c + D_a) [a_i (a_i^\dagger)^2 a_i + (a_i^\dagger)^2 (a_i)^2] + (a_i, \to b_i, a_i^\dagger \to b_i^\dagger) \\
&\quad - \frac{J_E}{4} \sum_i \left[a_i^\dagger a_i^2 b_i + a_i b_i^\dagger b_i^2 + (b_i^\dagger)^2 b_i a_i^\dagger + b_i^\dagger (a_i^\dagger)^2 a_i + 4 a_i^\dagger a_i b_i^\dagger b_i \right]
\end{aligned} \quad \text{(S3)}$$

To find the magnon spectrum, we consider the quadratic part $\mathcal{H}^{(2)}$, which is linear in S (except the term proportional to the field) and can be conveniently written in terms of a Nambu spinor $\Psi_{\boldsymbol{k}}$ in Fourier space.

$$\mathcal{H}^{(2)} = \sum_{\boldsymbol{k}} \Psi_{\boldsymbol{k}}^\dagger h^{(2)}(\boldsymbol{k}) \Psi_{\boldsymbol{k}}, \text{ where } \Psi_{\boldsymbol{k}} = \begin{pmatrix} a_{\boldsymbol{k}} & b_{\boldsymbol{k}} & a_{-\boldsymbol{k}}^\dagger & b_{-\boldsymbol{k}}^\dagger \end{pmatrix}^T,$$
$$h^{(2)}(\boldsymbol{k}) = \frac{1}{2} \begin{pmatrix} f_{\boldsymbol{k}} + g\mu_B B & 0 & \frac{(D_c - D_a)S}{2} & J_E S \\ 0 & f_{\boldsymbol{k}} - g\mu_B B & J_E S & \frac{(D_c - D_a)S}{2} \\ \frac{(D_c - D_a)S}{2} & J_E S & f_{\boldsymbol{k}} + g\mu_B B & 0 \\ J_E S & \frac{(D_c - D_a)S}{2} & 0 & f_{\boldsymbol{k}} - g\mu_B B \end{pmatrix} \quad \text{(S4)}$$

and we have defined

$$a_{\boldsymbol{k}} = \frac{1}{\sqrt{N}} \sum_i e^{i\boldsymbol{k}\cdot\mathbf{r}_i} a_i \,, \; b_{\boldsymbol{k}} = \frac{1}{\sqrt{N}} \sum_i e^{i\boldsymbol{k}\cdot\mathbf{r}_i} b_i \,, \text{ and}$$

$$f_{\boldsymbol{k}} = S\left[\gamma_{\boldsymbol{k}} + \frac{(D_c + D_a)}{2} + J_E\right] \text{ with } \gamma_{\boldsymbol{k}} = 2\sum_{\delta} J_{\delta}[1 - \cos(\boldsymbol{k}\cdot\boldsymbol{\delta})]. \tag{S5}$$

Finding the dispersion of the two magnon branches $\omega_\mu(\boldsymbol{k})$ (labeled by $\mu = \pm$) then amounts to diagonalizing the following dynamical matrix (that preserves bosonic commutation relations).

$$D(\boldsymbol{k}) = M\mathcal{H}^{(2)}(\boldsymbol{k}), \text{ where } M = \begin{pmatrix} 1 & 0 & 0 & 0 \\ 0 & 1 & 0 & 0 \\ 0 & 0 & -1 & 0 \\ 0 & 0 & 0 & -1 \end{pmatrix} \tag{S6}$$

On doing so, we may write the quadratic part in terms of the magnon creation operators $\alpha^\dagger_{\mu,\boldsymbol{k}}$ as

$$\mathcal{H}^{(2)} = \sum_{\boldsymbol{k},\mu=\pm} \omega_\mu(\boldsymbol{k}) \left[\alpha^\dagger_{\mu,\boldsymbol{k}}\alpha_{\mu,\boldsymbol{k}} + \frac{1}{2}\right], \text{ where} \tag{S7}$$

$$\omega_\pm^2(\boldsymbol{k}) = (g\mu_B B)^2 + S^2\left[(D_a + D_c)J_E + D_aD_c + (D_a + D_c + 2J_E)\gamma_{\boldsymbol{k}} + \gamma_{\boldsymbol{k}}^2\right] \pm S\Big[(J_ES)^2(D_a - D_c)^2 + (g\mu_B B)^2(D_a + D_c)(D_a + D_c + 4J_E) + 4(g\mu_B B)^2\{(D_a + D_c + 2J_E)\gamma_{\boldsymbol{k}} + \gamma_{\boldsymbol{k}}^2\}\Big]^{1/2} \tag{S8}$$

We note that setting $\boldsymbol{k} = 0$ in Eq. (S8) implies $\gamma_{\boldsymbol{k}} = 0$, and this allows us to recover the dispersions of the two uniform AFMR modes $\omega_\pm$ discussed in the main text.

Next, we turn to the magnon-scattering terms, which may be re-written in terms of the α_μ bosons, which reads (suppressing the μ index for clarity)

$$\mathcal{H}^{(4)} = \frac{1}{N}\sum_{\boldsymbol{k},\boldsymbol{q},\boldsymbol{p}} V^{(4)}_{\boldsymbol{k},\boldsymbol{q},\boldsymbol{p}}\alpha^\dagger_{\boldsymbol{k}}\alpha^\dagger_{\boldsymbol{q}}\alpha_{\boldsymbol{p}}\alpha_{\boldsymbol{k}+\boldsymbol{q}-\boldsymbol{p}} + \bar{V}^{(4)}_{\boldsymbol{k},\boldsymbol{q},\boldsymbol{p}}\alpha^\dagger_{\boldsymbol{k}}\alpha_{\boldsymbol{q}}\alpha_{\boldsymbol{p}}\alpha_{\boldsymbol{k}-\boldsymbol{q}-\boldsymbol{p}} + \text{H.c.}. \tag{S9}$$

The magnon-scattering terms can be grouped into magnon number-conserving terms like $V^{(4)}$, and number-non-conserving terms like $\bar{V}^{(4)}$. The result of the presence of these terms would be to redistribute the population of the magnons when the $\boldsymbol{k} = 0$ mode (AFMR mode) is excessively populated to reach a steady state. The transient dynamics is a complicated kinetic problem, so

we proceed by making the physically reasonable assumption that a non-equilibrium steady state is reached with a finite density of non-zero momentum magnons. The collective dynamics of these magnons is responsible for spin diffusion, and enhances the relaxation-rate of the NV^- centers placed proximate to the sample.

For simplicity, let us consider a single NV^- center placed at a distance d from the CrSBr sample. The relaxation rate of the NV^- is mainly due to magnetic noise caused by longitudinal collective modes, as the magnon gaps $\omega_\pm$ are much larger than the NV^- splitting Ω. Therefore, we restrict ourselves to considering magnetic noise at the NV^- from such collective magnon modes, which in turn determine the longitudinal spin-correlations $C_{\|}(\boldsymbol{k}, \Omega)$ in the steady state. The corresponding relaxation rate (due to a CrSBr sample at distance d) is given by

$$\Gamma_1(\Omega) \propto \int d^2k\, k^2 e^{-2kd}\, C_{\|}(\boldsymbol{k}, \Omega). \tag{S10}$$

The net relaxation rate may be obtained via adding the relaxation rate due to each layer, assuming the layers are weakly correlated.

If the longitudinal spin-density (set by the magnon-number) was conserved and the system was in equilibrium, we would expect $C_{\|}(\boldsymbol{k}, \Omega)$ to take a diffusive form at the longest length-scales and time-scales. While neither is precisely true for our problem, the energy-scale of non-conservation (set by $D_{a/c}$) is a thousand times smaller relative to the dominant energy-scales $J_{1/2/3}$, and we also assume that the sample is near equilibrium, so that the spin-density is approximately conserved. Therefore, we may write (via the fluctuation-dissipation theorem)

$$C_{\|}(\boldsymbol{k}, \Omega) = \frac{2k_B T}{\Omega} \mathrm{Im}[\chi(\boldsymbol{k}, \Omega)], \text{ where } \chi(\boldsymbol{k}, \Omega) = \frac{\chi(D_s k^2 + \gamma_s)}{-i\Omega + D_s k^2 + \gamma_s} \tag{S11}$$

is the dynamical susceptibility, D_s is the spin diffusion constant, and γ_s captures the deviation from spin-density conservation. Since the NV^- frequency Ω is the lowest energy-scale in the problem, we can set it to zero for analytical convenience. We find that the relaxation rate is given by

$$\Gamma_1 \propto \int d^2k\, k^2 e^{-2kd} C_{\|}(\boldsymbol{k}, \Omega \to 0) = \int d^2k\, k^2 e^{-2kd} \frac{2k_B T \chi}{D_s k^2 + \gamma_s} \xrightarrow{D_s \gg \gamma_s d^2} \frac{2k_B T \chi}{d^2 D_s} \tag{S12}$$

The last approximation is a good approximation if the net spin-density (along b axis) is conserved, i.e., the magnon number is conserved, i.e., $\gamma_s \to 0$.

All that remains to be determined is the functional dependence of χ and D_s on the magnon density n. To derive this, we first note that each AFMR mode has a finite longitudinal magnetization

density (approximately, $\mathbf{m} \propto \chi(\mathbf{n} \times \dot{\mathbf{n}})$ where $\mathbf{n}$ is the Neel vector), as shown in Fig. 2(b),(c) in the main text. This spin density $\mathbf{m} \cdot \hat{b}$ is directly proportional to the number density of magnons n, so we need to essentially determine the spin-susceptibility and diffusivity due to magnon dynamics. It is convenient to use the Einstein relation that the magnon conductivity $\sigma_s = \chi D_s$, then we find that $\Gamma_1 \sim \sigma_s D_s^{-2}$. Now, we note that within a semi-classical picture of magnon conduction, the magnon conductivity $\sigma_s \propto n\tau$, where n is the density and τ is the scattering time. If magnon-scattering dominates momentum relaxation, then $\tau \propto 1/n$, implying that σ_s is independent of the density. Physically, the effect of a larger number of carriers is canceled by the fact that the carriers also collide more frequently. On the other hand, the diffusion constant $D_s \sim \langle v^2 \rangle \tau \propto \langle v^2 \rangle / n$, where $\langle v^2 \rangle$ is the mean-squared velocity that is set by the temperature, so the diffusion constant decreases with increasing density n. Combining these two results, we find the density dependence of the NV^- relaxation rate to be $\Gamma_1 \sim \sigma_s D_s^{-2} \sim n^2$. Thus, we have shown that an increased number density of magnons, created by pumping, can cause faster relaxation of a proximate NV^- center.

Magnetization Configurations in an Applied Field

The two configurations of the antiferromagnetic sublattice magnetizations mentioned in the main text are those of the antiparallel ("below spin-flop") and canted ("spin-flop") geometries (see Fig. S4). In zero applied field, the equilibrium state of both antiferromagnets is the antiparallel state, with the sublattice magnetizations lying along an easy-axis as in Fig. S4A. In a magnetic field applied perpendicular to this axis the spins will cant in the direction of the field at arbitrarily small field, and this canting will increase with field asymptotically approaching the parallel or "spin-flip" state as shown in Fig. S4B.

The behavior is different when the magnetic field is applied parallel to the easy axis. In this case the sublattice magnetizations will remain antiparallel until the field reaches the spin-flop field $H_{\mathrm{SF}} \approx \sqrt{2H_E H_A}$, where H_E is the exchange field and H_A is the easy-axis anisotropy. Above H_{SF} the sublattice magnetizations abruptly reorient to the canted configuration and increasingly align with the applied field as the field increases. At an even higher field, the increasing contribution of the anisotropy causes the sublattice magnetizations to undergo another abrupt reorientation to fully align with the field in the parallel configuration shown in Fig. S4C.

In the case of $CrCl_3$ AFMR, because the in-plane anisotropy is negligible, the sublattice magnetizations reorient to the canted configuration of Fig S4B in the presence of even very small applied field. Since there is no in-plane easy axis, the sublattice magnetizations remain in this configuration at any finite field in our field range.

In contrast, for CrSBr the field is applied along the in-plane easy axis, and thus the sublattice magnetizations remain in the antiparallel configuration shown in Fig. S4A for all measurements, except for that shown in the inset to Fig. 3D where around 1500 G and 120 K we observe what may be antiferromagnetic resonance in the spin flop configuration of Fig. S4B. In all cases, antiferromagnetic resonance consists of small-angle precession of the sublattice magnetizations about these equilibrium directions.

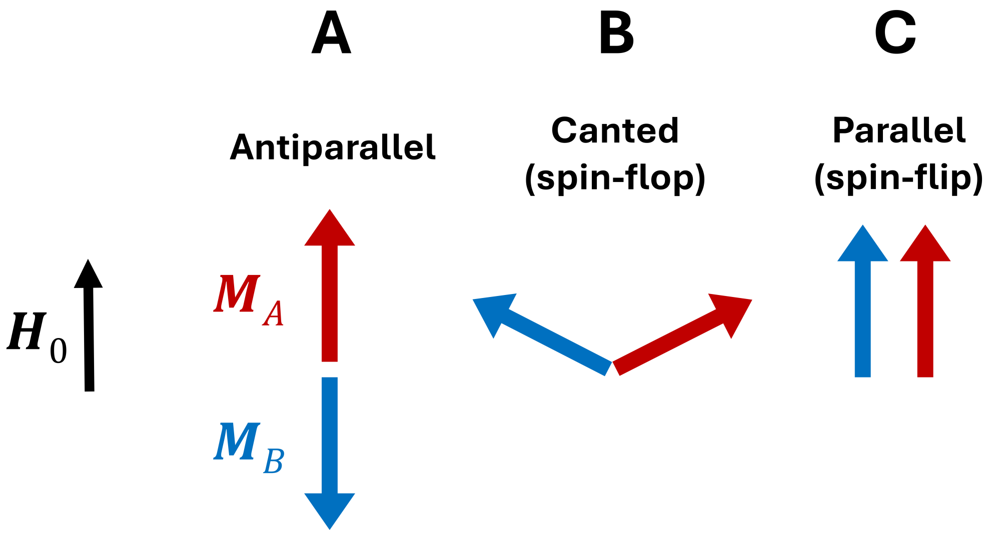

Figure S4: **Magnetization configurations for an antiferromagnet in an applied magnetic field.** Three configurations of the antiferromagnetic sublattice magnetizations for different strengths of field applied parallel to the anisotropy easy axis. Panel (A): From zero to low applied field H_0, the sublattice magnetizations remain aligned with the (here vertical) easy-axis and mutually antiparallel. Panel (B): At intermediate fields, the sublattice magnetizations lie in a canted configuration. Panel (C) At higher fields, the sublattice magnetizations lie parallel to their counterparts and to the applied field.

REFERENCES AND NOTES

1. V. Baltz, A. Manchon, M. Tsoi, T. Moriyama, T. Ono, Y. Tserkovnyak, Antiferromagnetic spintronics. *Rev. Mod. Phys.* **90**, 015005 (2018).

2. L. Šmejkal, Y. Mokrousov, B. Yan, A. H. MacDonald, Topological antiferromagnetic spintronics. *Nat. Phys.* **14**, 242–251 (2018).

3. B. Huang, G. Clark, D. R. Klein, R. Cheng, E. Navarro-Moratalla, K. L. Seyler, D. Zhong, E. Schmidgall, M. A. McGuire, D. H. Cobden, D. Xiao, W. Yao, P. Jarillo-Herrero, X. Xu, Layer-dependent ferromagnetism in a van der Waals crystal down to the monolayer limit. *Nature* **546**, 270–273 (2017).

4. B. Huang, G. Clark, D. R. Klein, D. MacNeill, E. Navarro-Moratalla, K. L. Seyler, N. Wilson, M. A. McGuire, D. H. Cobden, D. Xiao, W. Yao, P. Jarillo-Herrero, X. Xu, Electrical control of 2D magnetism in bilayer CrI_3. *Nat. Nanotechnol.* **13**, 544–548 (2018).

5. M. A. McGuire, H. Dixit, V. R. Cooper, B. C. Sales, Coupling of crystal structure and magnetism in the layered ferromagnetic insulator CrI_3. *Chem. Mater.* **27**, 612–620 (2015).

6. I. Lee, J. Cen, O. Molchanov, S. Feng, W. L. Huey, J. van Tol, J. E. Goldberger, N. Trivedi, H.-Y. Kee, P. C. Hammel, Spin-orbit coupling controlled two-dimensional magnetism in chromium trihalides. arXiv:2405.16709 [cond-mat.str-el] (2024).

7. D. MacNeill, J. T. Hou, D. R. Klein, P. Zhang, P. Jarillo-Herrero, L. Liu, Gigahertz frequency antiferromagnetic resonance and strong magnon-magnon coupling in the layered crystal $CrCl_3$. *Phys. Rev. Lett.* **123**, 047204 (2019).

8. T. M. J. Cham, S. Karimeddiny, A. H. Dismukes, X. Roy, D. Ralph, Y. K. Luo, Anisotropic gigahertz antiferromagnetic resonance of the easy-axis van der Waals antiferromagnet CrSBr. *Nano Lett.* **22**, 6716–6723 (2022).

9. J. Klein, T. Pham, J. D. Thomsen, J. B. Curtis, T. Denneulin, M. Lorke, M. Florian, A. Steinhoff, R. A. Wiscons, J. Luxa, Z. Sofer, F. Jahnke, P. Narang, F. M. Ross, Control of

structure and spin texture in the van der Waals layered magnet CrSBr. *Nat. Commun.* **13**, 5420 (2022).

10. W. Liu, X. Guo, J. Schwartz, H. Xie, N. U. Dhale, S. H. Sung, A. L. N. Kondusamy, X. Wang, H. Zhao, D. Berman, R. Hovden, L. Zhao, B. Lv, A three-stage magnetic phase transition revealed in ultrahigh-quality van der Waals bulk magnet CrSBr. *ACS Nano* **16**, 15917–15926 (2022).

11. X. Guo, W. Liu, J. Schwartz, S. H. Sung, D. Zhang, M. Shimizu, A. L. N. Kondusamy, L. Li, K. Sun, H. Deng, H. O. Jeschke, I. I. Mazin, R. Hovden, B. Lv, L. Zhao, Extraordinary phase transition revealed in a van der Waals antiferromagnet. *Nat. Commun.* **15**, 6472 (2024).

12. C. S. Wolfe, V. P. Bhallamudi, H. L. Wang, C. H. Du, S. Manuilov, R. M. Teeling-Smith, A. J. Berger, R. Adur, F. Y. Yang, P. C. Hammel, Off-resonant manipulation of spins in diamond via precessing magnetization of a proximal ferromagnet. *Phys. Rev. B* **89**, 180406(R) (2014).

13. T. van der Sar, F. Casola, R. Walsworth, A. Yacoby, Nanometre-scale probing of spin waves using single-electron spins. *Nat. Commun.* **6**, 7886 (2015).

14. C. Du, T. van der Sar, T. X. Zhou, P. Upadhyaya, F. Casola, H. Zhang, M. C. Onbasli, C. A. Ross, R. L. Walsworth, Y. Tserkovnyak, A. Yacoby, Control and local measurement of the spin chemical potential in a magnetic insulator. *Science* **357**, 195–198 (2017).

15. R. Schirhagl, K. Chang, M. Loretz, C. L. Degen, Nitrogen-vacancy centers in diamond: Nanoscale sensors for physics and biology. *Annu. Rev. Phys. Chem.* **65**, 83–105 (2014).

16. F. Jelezko, J. Wrachtrup, Single defect centres in diamond: A review. *Phys. Status Solidi A* **203**, 3207–3225 (2006).

17. A. Finco, A. Haykal, R. Tanos, F. Fabre, S. Chouaieb, W. Akhtar, I. Robert-Philip, W. Legrand, F. Ajejas, K. Bouzehouane, N. Reyren, T. Devolder, J.-P. Adam, J.-V. Kim, V. Cros, V. Jacques, Imaging non-collinear antiferromagnetic textures via single spin relaxometry. *Nat. Commun.* **12**, 767 (2021).

18. P. Vaidya, S. A. Morley, J. van Tol, Y. Liu, R. Cheng, A. Brataas, D. Lederman, E. del Barco, Subterahertz spin pumping from an insulating antiferromagnet. *Science* **368**, 160–165 (2020).

19. S. M. Rezende, A. Azevedo, R. L. Rodriguez-Suarez, Introduction to antiferromagnetic magnons. *J. Appl. Phys.* **126**, 151101 (2019).

20. C. M. Purser, V. P. Bhallamudi, F. Guo, M. R. Page, Q. Guo, G. D. Fuchs, P. C. Hammel, Spinwave detection by nitrogen-vacancy centers in diamond as a function of probe-sample separation. *Appl. Phys. Lett.* **116**, 202401 (2020).

21. L. Rondin, J.-P. Tetienne, T. Hingant, J.-F. Roch, P. Maletinsky, V. Jacques, Magnetometry with nitrogen-vacancy defects in diamond. *Rep. Prog. Phys.* **77**, 056503 (2014).

22. B. Flebus, Y. Tserkovnyak, Quantum-impurity relaxometry of magnetization dynamics. *Phys. Rev. Lett.* **121**, 187204 (2018).

23. B. A. McCullian, A. M. Thabt, B. A. Gray, A. L. Melendez, M. S. Wolf, V. L. Safonov, D. V. Pelekhov, V. P. Bhallamudi, M. R. Page, P. C. Hammel, Broadband multi-magnon relaxometry using a quantum spin sensor for high frequency ferromagnetic dynamics sensing. *Nat. Commun.* **11**, 5229 (2020).

24. H. Wang, S. Zhang, N. J. McLaughlin, B. Flebus, M. Huang, Y. Xiao, C. Liu, M. Wu, E. E. Fullerton, Y. Tserkovnyak, C. R. Du, Noninvasive measurements of spin transport properties of an antiferromagnetic insulator. *Sci. Adv.* **8**, eabg8562 (2022).

25. S. Chatterjee, J. F. Rodriguez-Nieva, E. Demler, Diagnosing phases of magnetic insulators via noise magnetometry with spin qubits. *Phys. Rev. B* **99**, 104425 (2019).

26. F. Machado, E. A. Demler, N. Y. Yao, S. Chatterjee, Quantum noise spectroscopy of dynamical critical phenomena. *Phys. Rev. Lett.* **131**, 070801 (2023).

27. Y. J. Bae, J. Wang, A. Scheie, J. Xu, D. G. Chica, G. M. Diederich, J. Cenker, M. E. Ziebel, Y. Bai, H. Ren, C. R. Dean, M. Delor, X. Xu, X. Roy, A. D. Kent, X. Zhu, Exciton-coupled coherent magnons in a 2D semiconductor. *Nature* **609**, 282–286 (2022).

28. Y. Sun, F. Meng, C. Lee, A. Soll, H. Zhang, R. Ramesh, J. Yao, Z. Sofer, J. Orenstein, Dipolar spin wave packet transport in a van der Waals antiferromagnet. *Nat. Phys.* **20**, 794–800 (2024).

29. T. C. Edwards, M. B. Steer, *Foundations for Microstrip Circuit Design* (John Wiley & Sons Ltd., 2016), chap. 7, pp. 157–199.

30. Y.-C. Chiang, H.-W. Tseng, C.-J. Yu, C.-Y. Lee, C.-C. Huang, C.-E. Ho, High-frequency signal transmission in a coplanar waveguide structure with different surface finishes. *Thin Solid Films* **784**, 140079 (2023).

31. M. Huang, J. C. Green, J. Zhou, V. Williams, S. Li, H. Lu, D. Djugba, H. Wang, B. Flebus, N. Ni, C. R. Du, Layer-dependent magnetism and spin fluctuations in atomically thin van der Waals magnet $CrPS_4$. *Nano Lett.* **23**, 8099–8105 (2023).

32. M. Huang, Z. Sun, G. Yan, H. Xie, N. Agarwal, G. Ye, S. H. Sung, H. Lu, J. Zhou, S. Yan, S. Tian, H. Lei, R. Hovden, R. He, H. Wang, L. Zhao, C. R. Du, Revealing intrinsic domains and fluctuations of moiré magnetism by a wide-field quantum microscope. *Nat. Commun.* **14**, 5259 (2023).

33. M. A. Tschudin, D. A. Broadway, P. Siegwolf, C. Schrader, E. J. Telford, B. Gross, J. Cox, A. E. E. Dubois, D. G. Chica, R. Rama-Eiroa, E. J. G. Santos, M. Poggio, M. E. Ziebel, C. R. Dean, X. Roy, P. Maletinsky, Imaging nanomagnetism and magnetic phase transitions in atomically thin CrSBr. *Nat. Commun.* **15**, 6005 (2024).

34. R. Xue, N. Maksimovic, P. E. Dolgirev, L.-Q. Xia, R. Kitagawa, A. Müller, F. Machado, D. R. Klein, D. MacNeill, K. Watanabe, T. Taniguchi, P. Jarillo-Herrero, M. D. Lukin, E. Demler, A. Yacoby, Signatures of magnon hydrodynamics in an atomically-thin ferromagnet. arXiv:2403.01057 [cond-mat.mes-hall] (2024).

35. M. E. Ziffer, F. Machado, B. Ursprung, A. Lozovoi, A. B. Tazi, Z. Yuan, M. E. Ziebel, T. Delord, N. Zeng, E. Telford, D. G. Chica, D. W. deQuilettes, X. Zhu, J. C. Hone, K. L. Shepard, X. Roy, N. P. de Leon, E. J. Davis, S. Chatterjee, C. A. Meriles, J. S. Owen, P. J.

Schuck, A. N. Pasupathy, Quantum noise spectroscopy of criticality in an atomically thin magnet. arXiv:2407.05614 [cond-mat.mes-hall] (2024).

36. S. Das, A. L. Melendez, I.-H. Kao, J. A. García-Monge, D. Russell, J. Li, K. Watanabe, T. Taniguchi, J. H. Edgar, J. Katoch, F. Yang, P. C. Hammel, S. Singh, Quantum sensing of spin dynamics using boron-vacancy centers in hexagonal boron nitride. *Phys. Rev. Lett.* **133**, 166704 (2024).

37. R. Gong, X. Du, E. Janzen, V. Liu, Z. Liu, G. He, B. Ye, T. Li, N. Y. Yao, J. H. Edgar, E. A. Henriksen, C. Zu, Isotope engineering for spin defects in van der Waals materials. *Nat. Commun.* **15**, 104 (2024).

38. K. Yang, G. Wang, L. Liu, D. Lu, H. Wu, Triaxial magnetic anisotropy in the two-dimensional ferromagnetic semiconductor CrSBr. *Phys. Rev. B* **104**, 144416 (2021).

39. A. Scheie, M. Ziebel, D. G. Chica, Y. J. Bae, X. Wang, A. I. Kolesnikov, X. Zhu, X. Roy, Spin waves and magnetic exchange Hamiltonian in CrSBr. *Adv. Sci.* **9**, 2202467 (2022).

40. A. Auerbach, *Interacting Electrons and Quantum Magnetism* (Springer Science & Business Media, 1998).
